# Effect of reduction in skeletal muscle quantity and sex differences on postoperative hepatic steatosis following total pancreatectomy

**DOI:** 10.1007/s00595-026-03340-x

**Published:** 2026-05-29

**Authors:** Mitsuhiro Shimura, Masamichi Mizuma, Shimpei Maeda, Fuyuhiko Motoi, Mika Ando, Yuichiro Umino, Hideaki Sato, Shuichi Aoki, Koetsu Inoue, Masahiro Iseki, Daisuke Douchi, Takayuki Miura, Masaharu Ishida, Takashi Kamei, Michiaki Unno

**Affiliations:** 1 Department of Surgery, Tohoku University Graduate School of Medicine, 1-1 Seiryomachi, Aoba-ku, Sendai, 980-8574 Japan; 2 Department of Gastroenterological, General, Breast and Thyroid Surgery, Yamagata University Graduate School of Medical Science, Yamagata, Japan

**Keywords:** Total pancreatectomy, Hepatic steatosis, Sex-specific risk factor, Body compositional change, Muscle loss

## Abstract

**Purpose:**

This study aimed to determine the risk factors for new-onset hepatic steatosis after total pancreatectomy (TP), focusing on muscle loss and sex.

**Methods:**

We retrospectively analyzed 100 patients who underwent TP between 2005 and 2024. Nutritional parameters, BMI, muscle volume, and liver status were evaluated. Logistic regression and subgroup analyses were performed to identify the risk factors for hepatic steatosis.

**Results:**

The prevalence of hepatic steatosis increased from 3.0% preoperatively to 24.0% at 3–6 months after TP. Female patients showed a significantly higher incidence of hepatic steatosis than male patients (37.8% vs. 12.7%, respectively). A multivariate analysis identified female sex (odds ratio: 7.77, 95% confidence interval: 2.03–29.8, *p* = 0.009), younger age (0.93, 0.88–0.98, 0.011), and higher preoperative BMI (1.28, 1.05–1.63, 0.015) as independent risk factors. Among the female patients, a younger age, one-stage TP, greater reductions in SMI, portal vein resection, longer operative time, greater blood loss, and diarrhea at discharge were associated with new-onset hepatic steatosis. However, a lower intraoperative blood loss potentially contributes to recovery from hepatic steatosis after TP.

**Conclusions:**

A female sex is a high risk factor for hepatic steatosis after TP. Reducing operative stress and preserving muscle volume may facilitate the prevention and recovery from hepatic steatosis.

**Supplementary Information:**

The online version contains supplementary material available at 10.1007/s00595-026-03340-x.

## Introduction

Total pancreatectomy (TP), particularly one-stage TP and total remnant pancreatectomy (TRP), results in complete pancreatic endocrine and exocrine insufficiencies [1–3]. Despite recent improvements in surgical techniques, TP still has a higher perioperative mortality rate than pancreaticoduodenectomy (PD) and distal pancreatectomy. Therefore, TP requires careful perioperative management [4]. Two recent randomized phase 3 trials for resectable pancreatic ductal adenocarcinoma (PDAC) showed that multimodal treatment approaches, incorporating neoadjuvant chemotherapy, surgery, and adjuvant chemotherapy, have become the standard strategy, resulting in an improved survival outcomes [5, 6]. Consequently, with the postoperative prognosis after the first pancreatectomy becoming prolonged, an increasing number of patients require TRP to manage local recurrence or metachronous lesions in the remnant pancreas [7, 8].

Several studies have shown that 19.6%–75.0% of patients develop hepatic steatosis after pancreatectomy within 1 year [9–19]. Additionally, previous studies have reported that the incidence of postoperative hepatic steatosis peaks approximately 6 months after pancreatectomy [14]. An emergent metabolic imbalance due to the absence of pancreatic enzymes and hormones, especially insulin, affects hepatic lipid accumulation [20–22]. Hepatic steatosis in adults is currently the most frequent cause of chronic liver disease worldwide, and its prevalence continues to increase [23]. Hepatic steatosis is generally more prevalent and severe in adult males than in adult females [23]. However, few studies have emphasized sex-specific differences in liver status following pancreatectomy, especially TP.

In parallel with the increasing attention paid to hepatic steatosis after pancreatectomy, there has been growing recognition of sarcopenia as a prognostic and metabolic determinant in patients with cancer [24, 25]. Skeletal muscle is the principal site of insulin-mediated glucose uptake. Accordingly, the loss of muscle mass promotes insulin resistance and may exacerbate hepatic steatosis through hyperinsulinemia and enhanced *de novo* lipogenesis [26]. However, the interaction between muscle loss and hepatic steatosis following TP, particularly regarding sex-specific differences, remains largely unknown.

Therefore, in this study, we retrospectively analyzed patients who underwent TP in the last two decades and assessed detailed changes in nutritional markers, body composition, and hepatic condition preoperatively and at 3–6 months and 1 year postoperatively. We aimed to identify risk factors for new-onset hepatic steatosis at 3–6 months after TP, with particular attention to sex-specific differences, and to examine the factors associated with recovery from hepatic steatosis by the first postoperative year.

## Materials and methods

### Patients and data collection

**Fig. 1:**
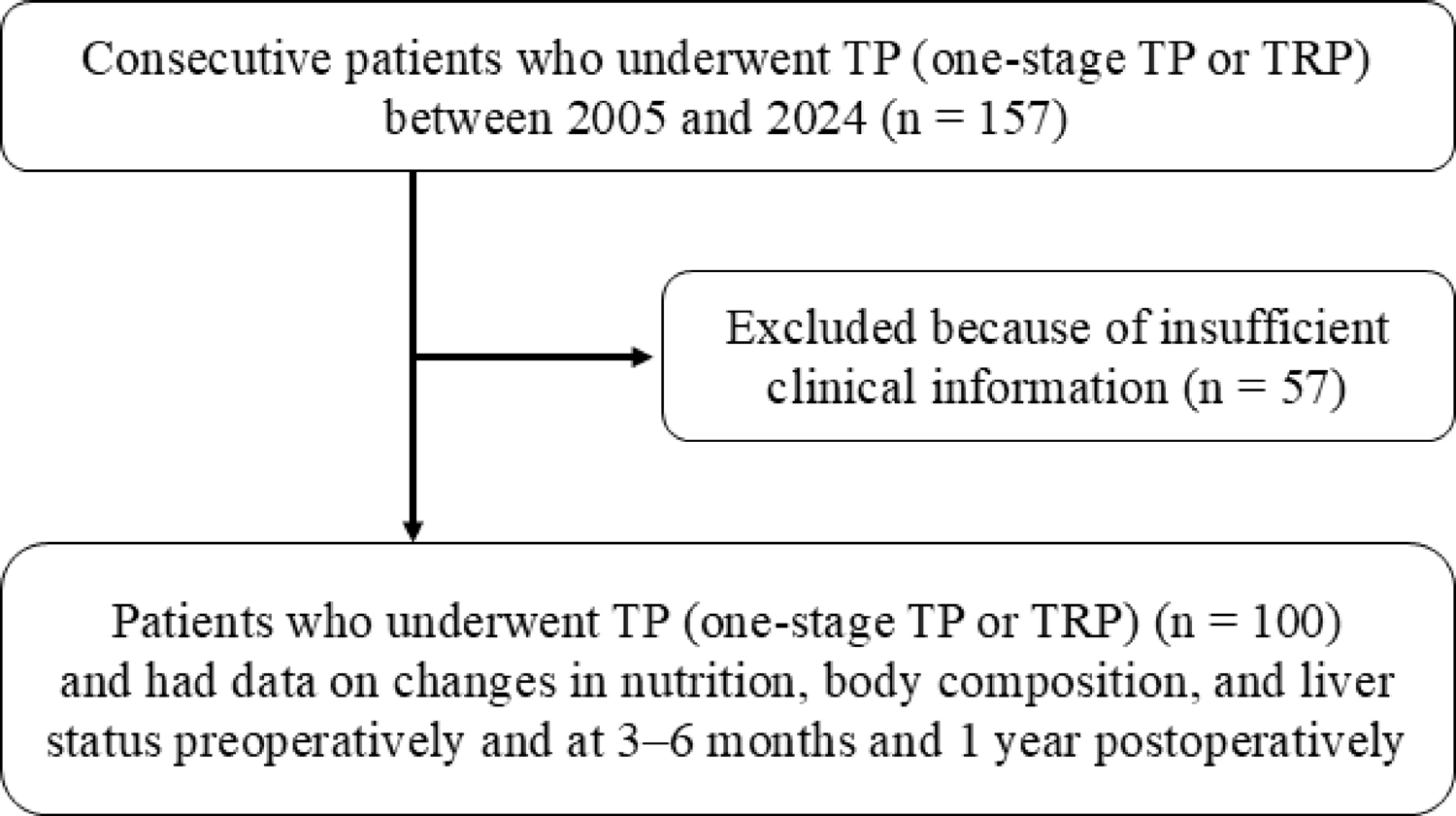
Flow chart of the patients included in the study. Total pancreatectomy, TP; total remnant pancreatectomy, TRP

We retrospectively reviewed 157 consecutive patients who underwent one-stage TP or TRP at Tohoku University Hospital between 2005 and 2024. Of these, 100 patients with complete clinical, laboratory, and radiological data preoperatively, at 3–6 months, and 1 year postoperatively were eligible for the analysis (Fig. 1). Changes in nutritional parameters, body composition, including the body mass index (BMI) and skeletal muscle mass index (SMI), and liver status were analyzed. The SMI and hepatic steatosis were evaluated using plain computed tomography (CT) images preoperatively and at 3–6 months and 1 year postoperatively. The SMI was calculated as the cross-sectional area of skeletal muscle (cm²) divided by height² (m²) at the L3 level, using blinded patient information. BMI was calculated from body weight measured on the same day as the CT examination for each corresponding time point. The reduction rates of BMI and SMI were defined as the postoperative value divided by the value at the reference time point, expressed as a percentage (%). Hepatic steatosis was defined as a CT density < 40 HU, which was equivalent to non-alcoholic fatty liver disease [10]. When multiple CT scans were available within 3–6 months, the image with the lowest liver attenuation value was selected for analysis. New-onset hepatic steatosis was defined as the absence of hepatic steatosis on the preoperative CT and its presence on the plain CT performed 3–6 months after TP. Recovery from hepatic steatosis was defined as a liver status in which hepatic steatosis was present on the plain CT at 3–6 months after TP, but improved to a CT value ≥ 40 HU on the plain CT at 1 year after TP. The ratio of aspartate aminotransferase (AST)/alanine aminotransferase (ALT) was measured as a marker of liver damage and fibrosis [10, 27]. The following clinical data were collected: pathological diagnosis; type of surgery; sex; age; whether there was recurrence; serum albumin (ALB) concentrations; Glasgow prognostic score; Controlling Nutritional Status score; serum HbA1c values; AST/ALT ratio; BMI; SMI; portal vein resection; superior mesenteric artery (SMA) nerve plexus dissection; duration of surgery; amount of blood loss; blood transfusion; early enteral nutrition via feeding tube; Clavien–Dindo classification; duration of the postoperative hospital stay; presence of diarrhea at discharge; postoperative supplementation with pancrelipase (Viatris Pharmaceutical Co., Ltd., Tokyo, Japan) and adjuvant chemotherapy. SMA nerve plexus dissection was recorded as a binary variable (performed vs. not performed). Perioperative glycemic control was managed by diabetes specialists, and intensive insulin therapy was initiated preoperatively or immediately after TP to achieve strict glycemic control in all patients. All patients received pancreatic enzyme replacement therapy (PERT) after TP. In Japan, pancrelipase was approved for insurance coverage in 2011. Before this approval, patients were prescribed other pancreatic enzyme preparations. Since 2011, pancrelipase has been primarily prescribed to all patients. Pancrelipase was generally administered at the full standard dose of 1800 mg/day.

### Statistical analysis

Data are presented as the median (interquartile range). Statistical analyses were performed using Prism version 10 (GraphPad Software, Boston, MA, USA) or JMP version 17.0 (SAS Institute, Cary, NC, USA). Statistical significance was calculated using Fisher’s exact and Pearson’s χ^2^ tests for categorical variables. Mann–Whitney U and t-tests were performed for continuous variables. Changes in ALB, HbA1c, AST/ALT, BMI, and SMI were analyzed using a mixed-effects model, followed by Dunnett’s multiple comparison test, with the preoperative values set as the reference control. Changes in the frequency of hepatic steatosis were analyzed using a generalized linear mixed-effects model. Multivariate analyses were performed using logistic regression to investigate the risk factors for new-onset hepatic steatosis after TP. Three patients with hepatic steatosis on preoperative CT images were excluded from the analysis of the risk factors for new-onset hepatic steatosis. Statistical significance was set at *p* < 0.05.

## Results

### Clinical characteristics of patients who underwent TP

The cohort consisted of 55 male and 45 female patients, indicating an almost equal sex distribution (Table 1). The median age was 68 years (range, 28–87 years). Regarding surgical procedures, 72 patients underwent one-stage TP, and 28 underwent TRP. The histopathological diagnoses included 51 cases of PDAC, and the detailed pathological diagnoses are shown in Table 2. The Controlling Nutritional Status classification indicated normal, light, moderate, and severe nutritional status in 43.4%, 39.4%, 15.2%, and 2.0% of patients, respectively. The preoperative prevalence of hepatic steatosis was 3.0% and was observed in only three patients (two men and one woman). BMI showed no sex-based difference (*p* = 0.357). However, SMI was significantly higher in male patients than in female patients (*p* < 0.001), indicating distinct and robust sex-specific differences in body composition.

**Table 1:** Clinical characteristics of 100 patients who underwent TP

Clinical factors	Values
Sex (male: female)	55: 45
Age (median (IQR))	68 (59–73)
One-stage TP: TRP	72: 28
PDAC: non-PDAC	51: 49
Preoperative ALB (g/dL, median (IQR))	3.7 (3.5–4.0)
Preoperative GPS (0: 1: 2, %)	69.7: 27.2: 3.0
Preoperative CONUT status (normal: light: moderate: severe, %)	43.4: 39.4: 15.2: 2.0
Preoperative HbA1c (NGSP, %, median (IQR))	6.6 (5.9–7.9)
Preoperative AST/ALT ratio	1.1 (0.8–1.3)
Preoperative hepatic steatosis (< 40 HU, %)	3 (3.0)
Preoperative BMI (kg/m^2^, median (IQR), male: female)	21.6 (19.8–24.3): 21.9 (20.1–24.3)
Preoperative SMI (cm^2^/m^2^, median (IQR), male: female)	41.1 (35.7–47.7): 33.9 (30.4–39.1)
Portal vein resection (%)	26 (26.0)
SMA nerve plexus dissection (%)	10 (10.0)
Operative time (minutes, median (IQR))	536 (459–650)
Operative blood loss (ml, median (IQR))	1145 (649–2211)
Blood transfusion (yes, %)	36 (36.0)
Early enteral nutrition via feeding tube (%)	73 (73.0)
Clavien–Dindo classification (> IIIa, %)	19 (19.0)
Postoperative hospital stay (days, median (IQR))	28 (22–37)
Diarrhea at discharge (%)	13 (13.0)
Pancrelipase administration at discharge (%)	78 (78.0)
Adjuvant chemotherapy (%)	53 (53.0)

ALB, albumin; ALT, alanine aminotransferase; AST, aspartate aminotransferase; BMI, body mass index; CONUT, Controlling Nutritional Status; GPS, Glasgow prognostic score; IQR, interquartile range; NGSP, National Glycohemoglobin Standardization Program; PDAC, pancreatic ductal adenocarcinoma; TP, total pancreatectomy; TRP, total remnant pancreatectomy; SD, standard deviation; SMA, Superior mesenteric artery; SMI, skeletal mass index

**Table 2:** Pathological diagnosis in TP and TRP

	TP (*n* = 72)	TRP (*n* = 28)
PDAC	37	14
IPMN/C	21	11
Pancreatic AVM	3	-
Metastasis of renal cancer	3	1
PanNEN	3	-
Chronic pancreatitis	1	-
IOPC	1	1
SCN	1	-
SPN	1	-
ITPN	1	-
High-grade PanIN	-	1

AVM, arteriovenous malformation; IOPC, intraductal oncocytic papillary carcinoma; IPMN/C, intraductal papillary mucinous neoplasm/carcinoma; ITPN, Intraductal tubulopapillary neoplasm; PanIN, pancreatic intraepithelial neoplasia; PanNEN, pancreatic neuroendocrine neoplasm; PDAC, pancreatic ductal adenocarcinoma; SCN, serous cystic neoplasm; SPN, solid pseudopapillary neoplasm; total pancreatectomy, TP; total remnant pancreatectomy, TRP

Regarding operative factors, portal vein resection and SMA nerve plexus dissection were performed in 26 (26.0%) and 10 (10.0%) patients, respectively. The median operative time and intraoperative blood loss were 536 (459–650) min (median, interquartile range (IQR)) and 1145 (649–2211) mL, respectively, indicating a highly invasive surgical approach. Thirteen (13.0%) patients required diarrheal medication at discharge. Of the 13 patients with diarrhea, 4 (30.8%) underwent SMA nerve plexus dissection, whereas among the 87 patients without diarrhea, 6 (6.9%) underwent SMA nerve plexus dissection. SMA nerve plexus dissection was significantly associated with diarrhea at discharge (*p* = 0.024). Pancrelipase as PERT was prescribed in 78 (78.0%) patients, and all patients received PERT after TP during hospitalization.

### Changes in nutritional parameters, body composition, and liver status within 1 year after TP

**Fig. 2:**
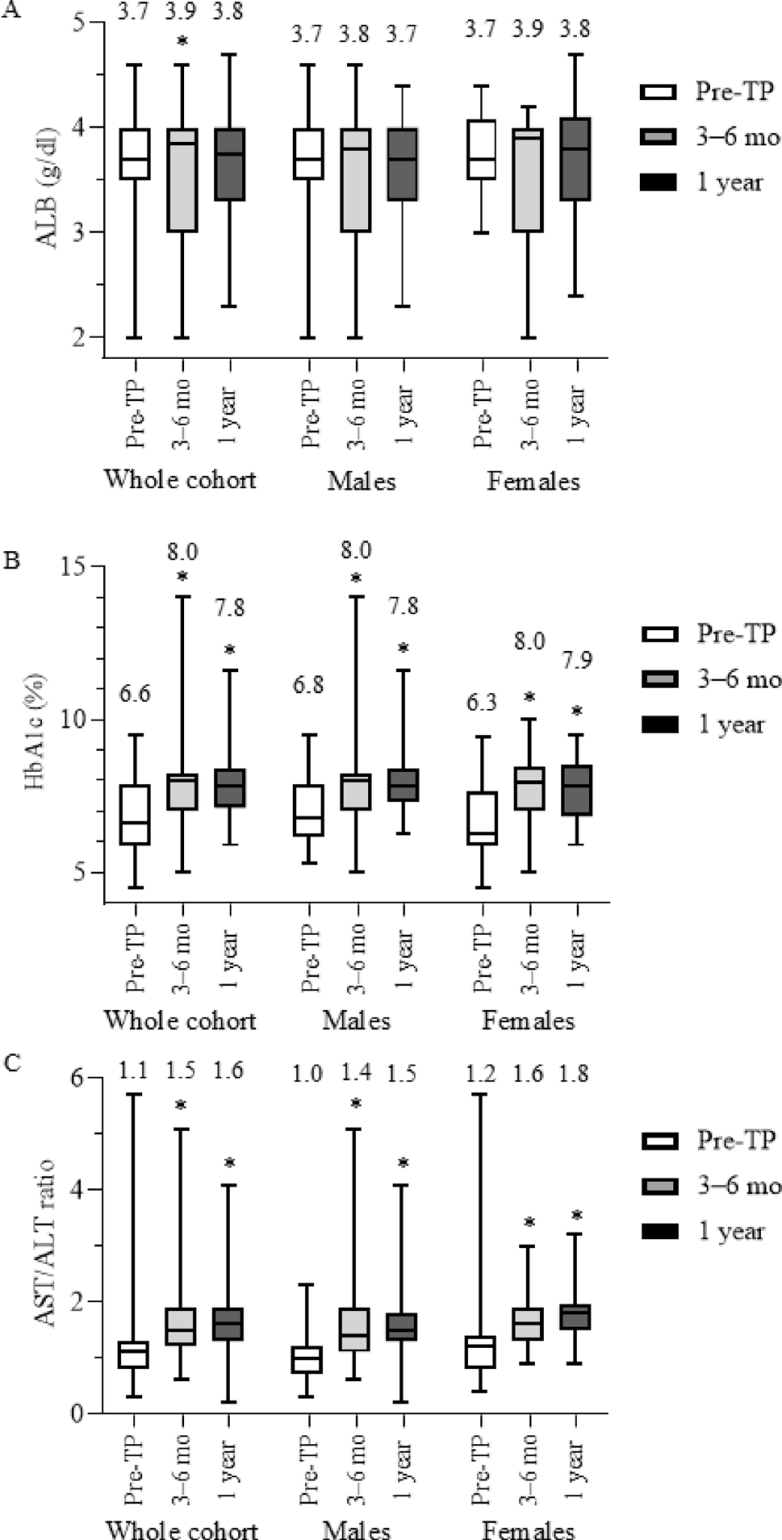
Sex-specific changes in the ALB concentrations, HbA1c values, and the AST/ALT ratio in patients after TP. (**A**) Serum ALB concentrations, (**B**) HbA1c values, and (**C**) the AST/ALT ratio. Each data point on the graph represents the median value from the following time points: pre-TP, 3–6 months post-TP, and 1 year post-TP. ALB, albumin; ALT, alanine aminotransferase; AST, aspartate aminotransferase; mo, months; y, year; TP, total pancreatectomy. *p < 0.05, preoperation vs. 3–6 months post-TP or preoperation vs. 1 year post-TP

Figure 2 shows the longitudinal changes in serum ALB concentrations, HbA1c values, and AST/ALT ratio (median values for each), stratified by all patients, male patients, and female patients. No obvious sex differences were observed in the longitudinal changes in serum ALB levels. HbA1c values significantly increased from 6.6% preoperatively to 8.0% at 3–6 months (*p* < 0.001) and to 7.8% at 1 year after TP (*p* < 0.001). This finding indicates a complete loss of pancreatic endocrine function. Similar trends in HbA1c levels were observed in both sexes. The AST/ALT ratio significantly increased over time, from 1.1 preoperatively to 1.5 at 3–6 months (*p* < 0.001) and 1.6 at 1 year postoperatively (*p* < 0.001). Additionally, similar trends in the AST/ALT ratio were observed in both sexes.

**Fig. 3:**
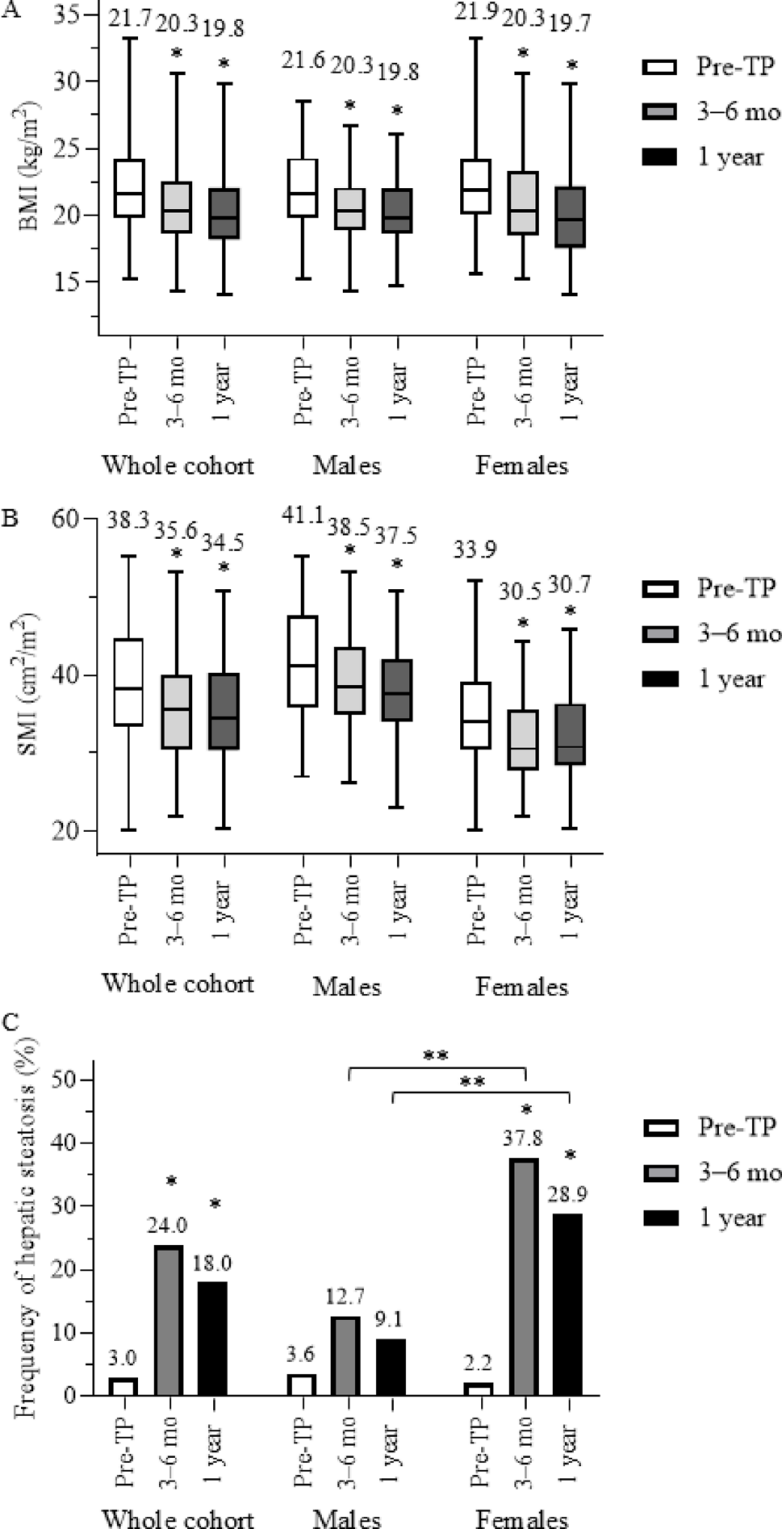
Sex-specific changes in body composition and the liver status of patients after TP. (**A**) BMI, (**B**) SMI, and (**C**) frequency of hepatic steatosis. Each data point on the graph represents the median value from the following time points: pre-TP, 3–6 months post-TP, and 1 year post-TP. BMI, body mass index; mo, months; y, year; TP, total pancreatectomy; SMI, skeletal mass index; hepatic steatosis, hepatic steatosis *p < 0.05, preoperation vs. 3–6 months post-TP or preoperation vs. 1 year post-TP; **p < 0.05, male vs. female patients

Figure 3 shows the longitudinal changes in the BMI, SMI, and the prevalence of hepatic steatosis, stratified by all patients, male patients, and female patients. In all groups, BMI was significantly decreased at 3–6 months and 1 year postoperatively compared with preoperative values (both *p* < 0.001). In male patients, BMI decreased from 21.6 kg/m^2^ preoperatively to 20.3 kg/m^2^ at 3–6 months (*p* < 0.001) and to 19.8 kg/m^2^ at 1 year (*p* < 0.001), and in female patients, it declined from 21.9 kg/m^2^ to 20.3 kg/m^2^ (*p* < 0.001) and 19.7 kg/m^2^ (*p* < 0.001), respectively. Similar to BMI, the SMI was significantly decreased in all groups at 3–6 months and 1 year postoperatively compared with preoperative values (both *p* < 0.001). In male patients, the SMI decreased from 41.1 cm^2^/m^2^ to 38.5 cm^2^/m^2^ (*p* < 0.001) and 37.5 cm^2^/m^2^ (*p* < 0.001), and in female patients, it decreased from 33.9 cm^2^/m^2^ to 30.5 cm^2^/m^2^ (*p* < 0.001) and 30.7 cm^2^/m^2^ (*p* = 0.007), at the preoperative, 3–6-month, and 1-year time points, respectively. The frequency of hepatic steatosis markedly increased by 3–6 months postoperatively (Fig. 3C). In the overall cohort, the prevalence of hepatic steatosis rose from 3.0% preoperatively to 24.0% at 3–6 months (*p* < 0.001) and then slightly improved to 18.0% at 1 year (*p* < 0.001), while a cumulative incidence curve showed that the incidence of new-onset hepatic steatosis gradually increased until approximately 6 months after TP, then plateaued (Supplementary Fig. 1). Notably, the frequency of hepatic steatosis in female patients significantly increased from 2.2% preoperatively to 37.8% at 3–6 months after TP compared with that in male patients who showed an increase from 3.6% to 12.7% (male patients: 12.7%; female patients: 37.8%; *p* = 0.004). Furthermore, this trend persisted for up to 1 year after TP (male patients: 9.1%; female patients: 28.9%; *p* = 0.010), which indicated a definite sex difference in the risk of hepatic steatosis after TP.

### Univariate and multivariate analyses of new-onset hepatic steatosis at 3–6 months after TP

In the univariate analysis, female sex, a younger age, one-stage TP, a higher preoperative BMI, a greater rate of reduction in SMI, portal vein resection, and diarrhea at the time of discharge were identified as risk factors associated with new-onset hepatic steatosis (Table 3). A multivariate logistic regression analysis using the variables selected in the univariate analysis showed that female sex (odds ratio: 7.77, 95% confidence interval: 2.03–29.8, *p* = 0.009), younger age (0.93, 0.88–0.98, 0.011), and higher preoperative BMI (1.28, 1.05–1.63, 0.015) were independent risk factors for the development of hepatic steatosis at 3–6 months after TP.

**Table 3:** Uni- and multivariate analyses of new-onset hepatic steatosis 3–6 months after TP (*n* = 97)

Variables	Univariate analysis			Multivariate analysis		
	Hepatic steatosis (*n* = 22)	Normal (*n* = 75)	*p*	Odds ratio	95% CI	*p*
Female (n, %)	16 (72.7)	28 (37.3)	**0.003**	7.77	2.03–29.8	**0.009**
Age (median (IQR))	61 (51–71)	69 (60–75)	**0.005**	0.93	0.88–0.98	**0.011**
One-stage TP (n, %)	20 (90.9)	50 (66.7)	**0.020**	1.56	0.25–9.71	0.629
PDAC (n, %)	12 (54.6)	37 (49.3)	0.426			
Preoperative ALB (g/dL, median (IQR))	3.7 (3.5–4.2)	3.7 (3.5–4.0)	0.324			
Preoperative HbA1c (NGSP, %, median (IQR))	6.6 (6.0–8.0)	6.6 (5.9–7.9)	0.312			
Preoperative AST/ALT ratio (median (IQR))	1.0 (0.7–1.3)	1.1 (0.8–1.4)	0.184			
Preoperative BMI (kg/m^2^, median (IQR))	23.2 (21.6–26.3)	21.4 (19.8–23.5)	**0.007**	1.28	1.05–1.63	**0.015**
Rate of reduction in BMI (%, median (IQR))	92.2 (85.9–98.6)	94.1 (90.1–98.5)	0.123			
Preoperative SMI (cm^2^/m^2^, median (IQR))	35.7 (32.9–45.5)	39.1 (33.3–44.1)	0.411			
Rate of reduction in SMI (%, median (IQR))	88.6 (77.7–95.6)	94.0 (84.5–102.3)	**0.006**	0.98	0.92–1.03	0.392
Portal vein resection (n, %)	10 (45.5)	15 (20.0)	**0.019**	1.38	0.31–6.06	0.675
SMA nerve plexus dissection (n, %)	3 (13.6)	6 (8.0)	0.332			
Operative time (minutes, median (IQR))	594 (500–697)	524 (458–641)	0.095			
Operative blood loss (ml, median (IQR))	1357 (1022–2278)	1050 (605–2042)	0.085			
Blood transfusion (yes, %)	10 (45.5)	24 (32.0)	0.181			
Early enteral nutrition via feeding tube (n, %)	16 (72.7)	56 (74.7)	0.527			
Clavien–Dindo classification (*>* IIIa, %)	5 (22.7)	13 (17.3)	0.385			
Diarrhea at discharge (n, %)	6 (27.3)	7 (9.3)	**0.041**	3.93	0.68–2270	0.125
Pancrelipase administration at discharge (n, %)	17 (77.3)	58 (77.3)	0.601			
Adjuvant chemotherapy (n, %)	15 (68.2)	36 (48.0)	0.076			

ALB, albumin; ALT, alanine aminotransferase; AST, aspartate aminotransferase; BMI, body mass index; IQR, interquartile range; NGSP, National Glycohemoglobin Standardization Program; PDAC, pancreatic ductal adenocarcinoma; TP, total pancreatectomy; SD, standard deviation; SMA, Superior mesenteric artery; SMI, skeletal mass index

### Subgroup analysis: female-specific risk factors for hepatic steatosis after TP and recovery-associated factors

Based on the finding that female sex was a particularly strong independent predictor of new-onset hepatic steatosis within 3–6 months after TP, we conducted a subgroup analysis to identify female-specific risk factors for the development of hepatic steatosis (Table 4). In female patients, the age was younger (*p* = 0.009) and one-stage TP (*p* = 0.030), frequency of portal vein resection (*p* = 0.005), operative time (*p* = 0.045), operative blood loss (*p* = 0.011), and frequency of diarrhea at the time of discharge (*p* = 0.004) were significantly higher in the hepatic steatosis group than in the normal group. Additionally, the hepatic steatosis group showed a greater percentage reduction in SMI (*p* = 0.002) than that in the normal group.

**Table 4:** Comparison of female patients with/without hepatic steatosis at 3–6 months after TP (*n* = 45)

Variables	Hepatic steatosis (*n* = 17)	Normal (*n* = 28)	*p*
Age (median (IQR))	61 (52–71)	72 (64–76)	**0.009**
One-stage TP (n, %)	15 (93.8)	18 (64.3)	**0.030**
PDAC (n, %)	9 (56.3)	16 (57.1)	0.600
Preoperative ALB (g/dL, median (IQR))	3.6 (3.5–4.0)	3.8 (3.5–4.1)	0.186
Preoperative HbA1c (NGSP, %, median (IQR))	6.2 (6.0–7.1)	6.6 (5.8–8.2)	0.302
Preoperative AST/ALT ratio (median (IQR))	1.2 (0.7–1.4)	1.2 (0.9–1.4)	0.140
Preoperative BMI (kg/m^2^, median (IQR))	22.6 (21.6–24.7)	21.3 (19.5–24.3)	0.131
Rate of reduction in BMI (%, median (IQR))	91.8 (85.6–97.7)	95.8 (91.9–97.7)	0.066
Preoperative SMI (cm^2^/m^2^, median (IQR))	35.0 (31.7–38.2)	33.2 (29.7–39.9)	0.320
Rate of reduction in SMI (%, median (IQR))	82.4 (77.0–91.2)	95.2 (87.9–103.9)	**0.002**
Portal vein resection (n, %)	9 (56.3)	4 (14.3)	**0.005**
Operative time (minutes, median (IQR))	570 (491–633)	470 (382–595)	**0.045**
Operative blood loss (ml, median (IQR))	1289 (973–2308)	656 (386–1855)	**0.011**
Blood transfusion (yes, %)	7 (43.8)	11 (39.3)	0.509
Clavien–Dindo classification (*>* IIIa, %)	4 (25.0)	2 (7.1)	0.116
Diarrhea at discharge (n, %)	5 (31.3)	0 (0)	**0.004**
Pancrelipase administration at discharge (n, %)	12 (75.0)	20 (71.4)	0.544

ALB, albumin; ALT, alanine aminotransferase; AST, aspartate aminotransferase; BMI, body mass index; IQR, interquartile range; NGSP, National Glycohemoglobin Standardization Program; PDAC, pancreatic ductal adenocarcinoma; TP, total pancreatectomy; SD, standard deviation; SMI, skeletal mass index

**Fig. 4:**
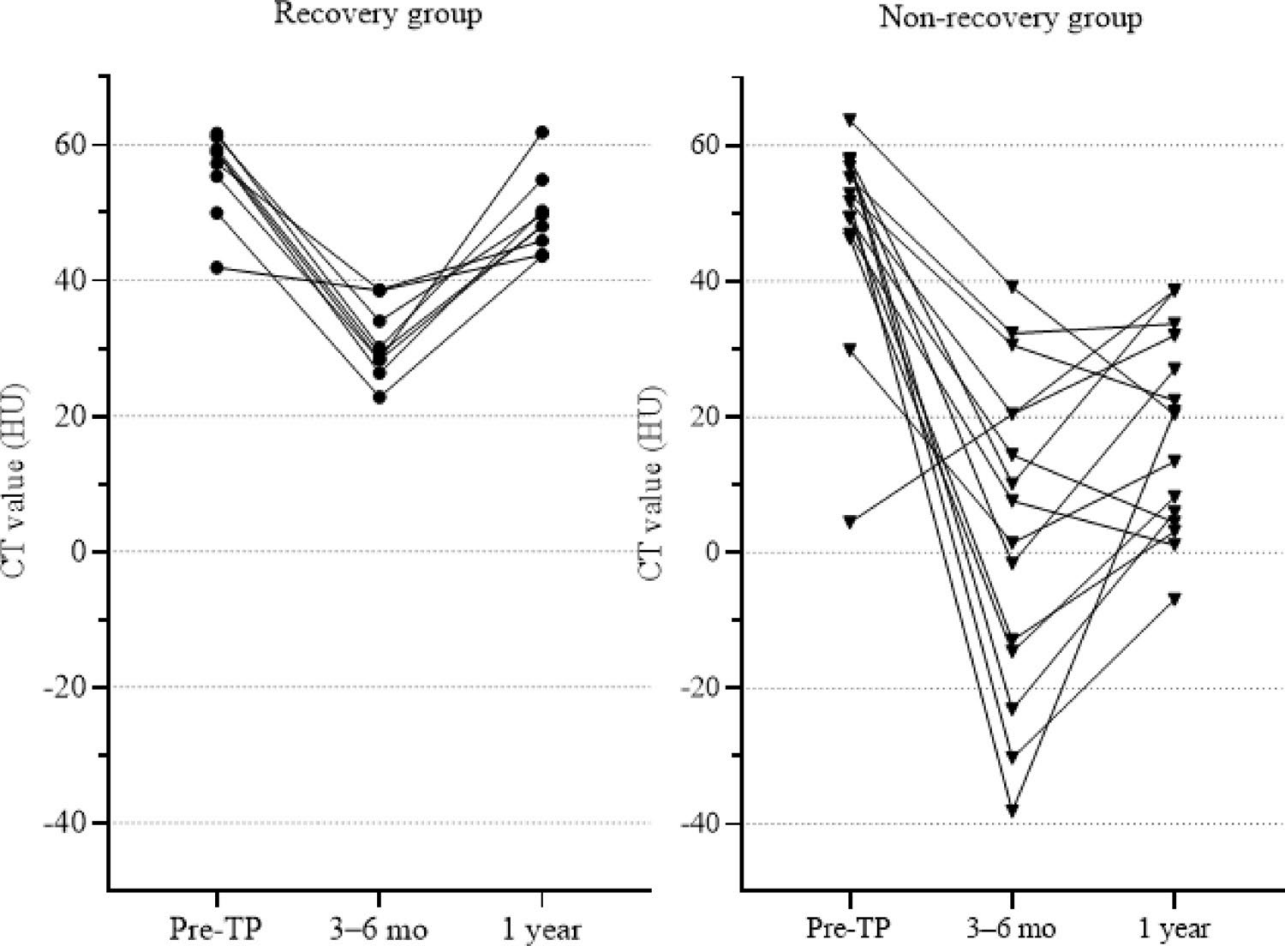
The attenuation and recovery of the CT value in the liver after TP**. **The left and right graphs illustrate the changes in liver CT values in the recovery and non-recovery groups, respectively. Hepatic steatosis was defined as a CT density of < 40 HU

Among the 24 patients who developed hepatic steatosis 3–6 months after TP, nine (37.5%) showed recovery from 3 to 6 months to 1 year after TP (Table 5; Fig. 4). Among the nutritional and body composition parameters, only a lower intraoperative blood loss was significant in the recovery group (*p* = 0.021). Portal vein resection was performed more frequently in the non-recovery group than in the recovery group (60.0% vs. 22.2%, *p* = 0.084). However, this difference was not significant, probably because of the small sample size.

**Table 5:** Recovery-associated factors for hepatic steatosis (*n* = 24)

Variables	Recovery (*n* = 9)	Non-recovery (*n* = 15)	*p*
Female sex (n, %)	6 (66.7)	11 (73.3)	0.539
Age (median (IQR))	62 (55–77)	60 (41–71)	0.084
PDAC (n, %)	3 (33.3)	11 (73.3)	0.067
One-stage TP (n, %)	8 (88.9)	13 (86.7)	0.692
Portal vein resection (n, %)	2 (22.2)	9 (60.0)	0.084
Operative time (minutes, median (IQR))	566 (434–750)	622 (505–694)	0.456
Operative blood loss (ml, median (IQR))	1026 (866–1293)	2240 (1120–4385)	**0.021**
Blood transfusion (yes, %)	3 (33.3)	8 (53.3)	0.300
Clavien–Dindo classification (*>* IIIa, %)	3 (33.3)	3 (20.0)	0.397
Postoperative hospital stays (days, median (IQR))	29 (24–39)	29 (24–32)	0.375
Diarrhea at the time of discharge (%)	1 (11.1)	5 (33.3)	0.238
Pancrelipase administration at the time of discharge (%)	6 (66.7)	13 (86.7)	0.255
Recurrence within 1 year after TP (n, %)	4 (44.4)	5 (33.3)	0.453
Preoperative ALB (g/dL, median (IQR))	3.6 (3.5–4.2)	3.8 (3.4–4.0)	0.445
Preoperative AST/ALT ratio (median (IQR))	0.9 (0.7–1.5)	1.0 (0.7–1.2)	0.377
Preoperative BMI (kg/m^2^, median (IQR))	23.3 (22.0–26.4)	22.3 (19.3–26.3)	0.288
BMI 3–6 months after TP (kg/m^2^, median (IQR))	21.6 (20.1–25.4)	21.7 (17.7–24.1)	0.225
BMI 1 year after TP (kg/m^2^, median (IQR))	19.7 (17.7–22.1)	19.0 (17.2–22.1)	0.463
Preoperative SMI (cm^2^/m^2^, median (IQR))	35.8 (34.1–46.7)	35.5 (30.4–45.1)	0.184
SMI 3–6 months after TP (cm^2^/m^2^, median (IQR))	32.7 (30.0–40.3)	29.5 (25.9–35.1)	0.130
SMI 1 year after TP (cm^2^/m^2^, median (IQR))	31.9 (24.8–43.2)	29.3 (26.6–34.2)	0.179
Rate of reduction in BMI (3–6 months/pre-TP, %, median (IQR))	91.5 (86.6–100.6)	94.3 (84.8–97.6)	0.401
Rate of reduction in BMI (1 year/3–6 months, %, median (IQR))	93.1 (80.1–97.7)	95.0 (89.4–100.0)	0.107
Rate of reduction in SMI (3–6 months/pre-TP, %, median (IQR))	90.9 (77.4–95.0)	86.9. (79.4–99.2)	0.432
Rate of reduction in SMI (1 year/3–6 months, %, median (IQR))	95.3 (86.0–107.0)	97.5 (87.8–107.9)	0.424

This analysis includes two patients who showed hepatic steatosis preoperatively and at 3–6 months after TP but recovered to a normal liver status at 1 year postoperatively

ALB, albumin; ALT, alanine aminotransferase; AST, aspartate aminotransferase; BMI, body mass index; IQR, interquartile range; NGSP, National Glycohemoglobin Standardization Program; PDAC, pancreatic ductal adenocarcinoma; TP, total pancreatectomy; SMI, skeletal mass index

## Discussion

This single-center, retrospective study provides novel insights into TP-induced hepatic steatosis. To the best of our knowledge, this study is the first to focus on the relationship between sex-specific body changes, especially SMI, and the new onset of hepatic steatosis after TP. We found that female sex, younger age, and higher preoperative BMI were independent risk factors for the development of hepatic steatosis after TP. In particular, female sex was the most significant risk factor for pancreatectomy-induced hepatic steatosis. Furthermore, we found that female-specific risk factors for the development of hepatic steatosis after TP were younger age, one-stage TP, a greater rate of reduction in SMI, portal vein resection, longer operative time, greater blood loss, and diarrhea at discharge. Lower blood loss during TP may improve hepatic steatosis. These findings suggest that in female patients, minimizing surgical invasiveness and providing meticulous management of postoperative diarrhea contribute to preserving body weight and skeletal muscle mass, thereby potentially preventing the development of hepatic steatosis following TP.

To date, studies on the risk factors for hepatic steatosis following pancreatectomy have been reported mainly from Asia, particularly Japan [13]. The following factors have been identified as potential risk factors: female sex [9, 10, 13, 16], high preoperative BMI [9, 15, 28], small remnant pancreas volume [15, 16, 28], pancreatic exocrine insufficiency [10, 15], PDAC [13, 15], postoperative weight loss [14, 15, 17], diarrhea [9, 10], older age [18] or younger age [15], high AST/ALT ratio [28], portal vein resection or superior mesenteric vein resection [13], longer operative time [28], and adjuvant chemotherapy [13]. In this study, consistent with previous reports, female sex and a higher preoperative BMI were identified as independent risk factors for new-onset hepatic steatosis. Moreover, one-stage TP, a higher rate of reduction in SMI, and chronic diarrhea were potential risk factors for the development of hepatic steatosis after TP in female patients.

The development of hepatic steatosis in adult females has been attributed to decreased estrogen concentrations after menopause [12, 13]. However, in this study, a younger age was identified as a risk factor for postoperative hepatic steatosis. Taken together with previous reports, a clear consensus has not yet been reached regarding the effect of age on the development of hepatic steatosis after pancreatic resection. Notably, a large-scale Korean follow-up study reported that high relative handgrip strength suppressed the development of hepatic steatosis in women but not in men [29]. However, a recent study investigating post-PD hepatic steatosis in patients with PDAC showed no significant association between preoperative sarcopenia and the development of hepatic steatosis postoperatively [15]. Similarly, in this study, preoperative SMI was not associated with the development of hepatic steatosis after TP. These findings suggest that the postoperative loss of muscle mass and strength in women may contribute to the development of hepatic steatosis.

In adults, hepatic steatosis development is generally influenced by lifestyle factors and overnutrition [23]. In contrast, post-pancreatectomy hepatic steatosis is often characterized by malnutrition resulting from pancreatic exocrine and endocrine insufficiency, suggesting a fundamentally different pathophysiological mechanism than that in adults without pancreatectomy. Although a low AST/ALT ratio (higher ALT/AST ratio) is recognized as a risk factor for hepatic steatosis in the adult population without pancreatectomy [30, 31], a high AST/ALT ratio appears to be a risk factor for hepatic steatosis after pancreatectomy [28]. We found a consistent increase in the AST/ALT ratio up to 1 year after TP in both sexes, suggesting that the mechanism of hepatic steatosis development differs from that observed in typical metabolic conditions.

Only a few studies have investigated the factors associated with recovery from hepatic steatosis after pancreatic resection. A small main pancreatic duct diameter, low serum amylase concentrations on postoperative day 28, and a high minimum liver CT value were reported as predictive factors for recovery from post-PD hepatic steatosis [28]. To the best of our knowledge, our study is the first to evaluate the factors associated with recovery from hepatic steatosis after TP. We found that patients with a greater blood loss showed a delayed recovery from hepatic steatosis. These results suggest that refining surgical techniques and reducing intraoperative invasiveness improve postoperative hepatic outcomes and facilitate recovery from hepatic steatosis after TP. Furthermore, the risk of new-onset hepatic steatosis differs according to the type of pancreatic resection, with the highest risk after TP, followed by PD and then distal pancreatectomy, supporting the notion that pancreatic exocrine insufficiency is a contributing factor to the development of hepatic steatosis [19, 32]. Several studies have reported that PERT is a crucial factor for improving or preventing hepatic steatosis after pancreatic resection [10, 19, 33]. However, a clear consensus regarding the efficacy of PERT for improving or preventing hepatic steatosis has not been established. A randomized, controlled trial failed to demonstrate a significant benefit of pancrelipase [18], and a recent meta-analysis similarly concluded that PERT was not associated with hepatic steatosis [13]. In this study, no significant association was observed between pancrelipase administration and the new development of hepatic steatosis. Nevertheless, because post-pancreatectomy hepatic steatosis has been reported to worsen the tolerance to gemcitabine-based adjuvant chemotherapy [17], adequate and early initiation of pancrelipase should still be recommended after TP. Furthermore, the latest research revealed that hepatic steatosis significantly accelerates PDAC progression and liver metastases through the migration inhibitory factor-CD44 axis, which indicates the importance of managing hepatic steatosis in PDAC patients [34].

This study has several limitations. First, this study was retrospective and conducted at a single center; nevertheless, it represents the largest cohort of patients who underwent TP at a single institution to date. Second, approximately one-third of the initially identified cases (*n* = 57) were excluded because of insufficient record information, which may have affected the results and limited the generalizability of the findings of this study. Third, we were unable to determine the detailed mechanisms underlying the risk factors associated with the new onset of hepatic steatosis in female patients. Finally, the number of cases analyzed for recovery-associated factors was small in this study. Therefore, further studies evaluating perioperative sex hormone status, particularly estrogen levels, nutritional malabsorption, skeletal muscle quantity and quality, and potential ethnic differences in baseline muscle mass are warranted to elucidate the mechanisms underlying the increased risk of new-onset hepatic steatosis after total pancreatectomy, particularly in female patients.

In summary, this study shows that a female sex, younger age, and higher preoperative BMI are independent risk factors for the new onset of hepatic steatosis after TP, and female patients show a particularly high risk. In female patients, surgical invasiveness, such as portal vein resection, a longer operative time, and a greater blood loss, might be associated with the new onset of hepatic steatosis, whereas reducing intraoperative blood loss may potentially lead to an earlier recovery from hepatic steatosis. Importantly, careful management of diarrhea and postoperative body compositional attenuation, particularly skeletal muscle loss, may be therapeutic targets for hepatic steatosis after TP.

## Supplementary Information

Below is the link to the electronic supplementary material.

Supplementary Material 1: Cumulative incidence of new-onset hepatic steatosis after TP. The cumulative incidence curve showed that the incidence of new-onset hepatic steatosis gradually increased until approximately 6 months after TP and then plateaued.TP, total pancreatectomy.
